# Systematic Review of the Measurement Properties of Tools Used to Measure Behaviour Problems in Young Children with Autism

**DOI:** 10.1371/journal.pone.0144649

**Published:** 2015-12-14

**Authors:** Jennifer Hanratty, Nuala Livingstone, Shannon Robalino, Caroline B. Terwee, Magdalena Glod, Inalegwu P. Oono, Jacqui Rodgers, Geraldine Macdonald, Helen McConachie

**Affiliations:** 1 School of Sociology, Social Policy and Social Work, Queen’s University Belfast, Belfast, Northern Ireland, United Kingdom; 2 Institute of Health and Society, Newcastle University, Newcastle upon Tyne, United Kingdom; 3 Department of Epidemiology and Biostatistics, VU University Medical Center, Amsterdam, The Netherlands; 4 Institute of Neuroscience, Newcastle University, Newcastle upon Tyne, United Kingdom; Vanderbilt University, UNITED STATES

## Abstract

**Background:**

Behaviour problems are common in young children with autism spectrum disorder (ASD). There are many different tools used to measure behavior problems but little is known about their validity for the population.

**Objectives:**

To evaluate the measurement properties of behaviour problems tools used in evaluation of intervention or observational research studies with children with ASD up to the age of six years.

**Methods:**

Behaviour measurement tools were identified as part of a larger, two stage, systematic review. First, sixteen major electronic databases, as well as grey literature and research registers were searched, and tools used listed and categorized. Second, using methodological filters, we searched for articles examining the measurement properties of the tools in use with young children with ASD in ERIC, MEDLINE, EMBASE, CINAHL, and PsycINFO. The quality of these papers was then evaluated using the COSMIN checklist.

**Results:**

We identified twelve tools which had been used to measure behaviour problems in young children with ASD, and fifteen studies which investigated the measurement properties of six of these tools. There was no evidence available for the remaining six tools. Two questionnaires were found to be the most robust in their measurement properties, the Child Behavior Checklist and the Home Situations Questionnaire—Pervasive Developmental Disorders version.

**Conclusions:**

We found patchy evidence on reliability and validity, for only a few of the tools used to measure behaviour problems in young children with ASD. More systematic research is required on measurement properties of tools for use in this population, in particular to establish responsiveness to change which is essential in measurement of outcomes of intervention.

**PROSPERO Registration Number:**

CRD42012002223

## Introduction

There is burgeoning research on how to improve the developmental progress and outcomes for young children with autism spectrum disorder (ASD) [1–3]. However, the field is held back by the multiplicity of ways of measuring outcomes [4]. Measurement often focuses on core diagnostic symptoms of autism, or on other characteristics which are expected to be relatively stable such as IQ, without sufficient attention to the question of how sensitive the tool may be in measuring change. Many measurement tools used in intervention and longitudinal observation studies may not have been specifically validated for use with children with ASD, particularly those tools which measure important determinants such as co-occurring conditions.

This paper focuses on one such co-occurring condition, behaviour problems in children. In this review, behaviour problems were defined as any behaviours that create problems for or challenge the individual and/or those who take care of them. These include behaviours that are not specific to autism, such as aggression, temper tantrums, non-compliance, as well as more specific problems, such as self-injury and eating non-food substances. Estimates of the prevalence of behaviour problems in ASD vary with the age range studied and the approach used. Using the Child Behavior Checklist (CBCL [5]), Hartley and colleagues [6] found one third of 169 children with ASD aged 1.5 to 5.8 years had total problem scores in the clinically significant range. Such behaviours take a heavy toll on families [7].

The purpose of this paper is to evaluate the measurement properties of tools used in research studies to measure behaviour problems in children with ASD aged up to 6 years. It builds on a large systematic review [8] commissioned in the UK to examine the available evidence on the measurement properties of tools used to measure progress and outcomes in young children with ASD. That review was conducted in two stages. The first was to identify the range of tools used in observational and intervention evaluation studies to measure outcomes for children. The second was to systematically search for and review papers about the measurement properties of those tools when used with young children with autism. The present paper evaluates information on tools used to measure behaviour problems, taken from this larger review, and extends it by updating the searches.

The review extends earlier work undertaken by the US Autism Speaks Foundation, both in scope and the quality of the evidence. In 2011, the Foundation established expert work groups to evaluate outcome measurement tools in three subdomains: restricted interests and repetitive behaviours [9], anxiety [10], and social communication behaviours [11]. The purpose was to identify tools appropriate for use in medication trials. Tools used in treatment trials of medication, complementary medicine or behavioural interventions, from 2005 to 2012, across any age group of children and youth with ASD, were identified through systematic searches. Other tools known to members of the work groups were also included. The tools were rated as: appropriate, appropriate with conditions, potentially appropriate/promising, unproven or not appropriate. The definitions of each level included information on reliability, validity and sensitivity to change of the tool, use with individuals with ASD, and also aspects of burden in terms of the time and other difficulties associated with use of the tool in assessment. In each case, a small number of tools were identified as “appropriate with conditions” (such as restricted age range, or lack of information on sensitivity to change).

The review on which this paper is based was broader in scope (progress and outcomes) though narrower in age range than the Autism Speaks Foundation work. We extended the search to include all studies published from 1992 to coincide with the publication of the international classifications, ICD-10 and DSM-IV [12, 13]. Further, our inclusion criteria were not confined to measures to be used in medication trials. Further, the measurement properties and appropriateness of a tool vary depending on the use to which the tool will be put. In a randomised trial of early intervention in ASD, for example, it is important to identify a primary outcome that can be assessed ‘blind’ and is responsive to change. In contrast, when monitoring children’s progress in a nursery setting, properties of face validity and content validity in relation to ASD, test-retest and inter-rater reliability, and measurement burden (cost, training, time) will assume greater importance. The review was registered with PROSPERO Registration Number: CRD42012002223

### Development of the review framework

Before examining how best to measure something, it is important to know what is important. Before conducting our searches, we therefore undertook consultations with groups of parents and young people with ASD, and a survey of early years professionals’ practice, to ascertain what each group of stakeholders considered important to measure by McConachie et al 2015 [8]. Using the findings from these consultations, together with developmental theory and the International Classification of Functioning [14], we developed a conceptual framework which we used to group 22 sub-domains of measurement tools. Behaviour Problems was one of these. Further consultation was undertaken at the end of the large review, bringing together a range of stakeholders (parents, young people with ASD, educationists, clinicians, researchers) for a Discussion Day. A selection of tools with some positive evidence about their measurement properties were looked at in detail by participants, and their views inform some conclusions drawn in this paper.

Preferred Reporting Items for Systematic Reviews and Meta-Analyses (PRISMA) standards are followed in this report (see S1 PRISMA Checklist).

## Stage 1: Identifying Tools Used to Measure Outcomes in Young Children with ASD

### Inclusion criteria—Stage 1

Our inclusion criteria were as follows [8]:

#### Types of studies

We included all randomised and quasi-randomised trials of social, psychological and educational early interventions for children with a diagnosis of ASD; observational studies of children with ASD (cross-sectional and longitudinal); case-control studies, and cohort studies, including studies of baby siblings of children with autism, which provide information on tools to monitor developmental progress and follow early markers of ASD.

#### Types of participants

Studies of children aged 0–6 years (at study entry) where at least 50% children were either diagnosed as having ASD or were being monitored for ASD symptoms. ASD was defined in terms of child participants having a ‘best estimate’ clinical diagnosis of an ASD, including autism, ASD, atypical autism, Asperger syndrome, and PDD-NOS, according to either ICD-10 or DSM-IV [12, 13] criteria. Use of a particular diagnostic tool such as the Autism Diagnostic Observation Schedule (ADOS) [15] or the Autism Diagnostic Interview (ADI-R) [16] was not required. Children with ASD and another health or mental health condition were included.

#### Types of measurement

We included the following types of measures:

- Direct assessment of ASD symptoms by a trained assessor
- Direct assessment of developmental skills, such as language, cognition, play skills, by a trained assessor
- Observational coding of social interaction skills
- Interview or self-completed (parent, teacher or other professional) questionnaire about child ASD symptoms.
- Interview or questionnaire about developmental skills, i.e. language (vocabulary), adaptive functioning, with/by parent, teacher or other professional.
- Interview or self-completed (parent, teacher or other professional) questionnaire about associated problems: including sleep, eating, toileting, anxiety, hyperactivity, behaviour that challenges, aggression, and others identified through parent consultation
- Idiographic measures focussed on particular behaviours (e.g. goal attainment scaling, target behaviours)
- Measures of impact on parent or family.

We excluded measures of economic impact on home and family, experimental tasks and measures (e.g. barrier tasks, reaction time, biophysical measures), medical investigations, or process measures (e.g. fidelity, adherence, parent satisfaction with intervention).

### Search strategy—Stage 1

We first searched the literature in June and July 2012, and updated this in June and July 2013 (last search conducted on 17^th^ July 2013). A master search strategy was created and modified as needed for searching across the databases. Modifications included changes to syntax, fields searched, and MeSH/thesaurus terms. A list of terms can be found in S1 Table ***Search Strategy*** along with an example search strategy for MEDLINE. Full search strategies are available from the corresponding author. Searches were limited to English language articles only. Where possible, search filters were used to limit study types returned.

The following databases were searched: Applied Social Sciences Index and Abstracts (ASSIA); Cumulative Index of Nursing and Allied Health (CINAHL); the Cochrane Library (includes DARE, HTA, CENTRAL, CDSR); ERIC; MEDLINE (including in-process and non-indexed); EMBASE; PsycINFO; Sociological Abstracts; Linguistics and Language Behavior Abstracts; Health Management Information Consortium (HMIC); PapersFirst (OCLC); Proceedings (OCLC); SCOPUS; Social Services Abstracts; Web of Science (Science Citation Index, Soc Sci Citation Index, Arts & Humanities Citation Index & Conference Proceedings Citation Index); WorldCatDissertations (OCLC).

Additionally, grey literature was searched via Digital Education Resource Archive (DERA), Oxford Patient Reported Outcomes Measurement Database, TRIP Database, internet searches, and searching of selected websites. The National Research Register and UK Clinical Research Network were also searched for ongoing studies.

### Selection of studies—Stage 1

**Fig 1:**
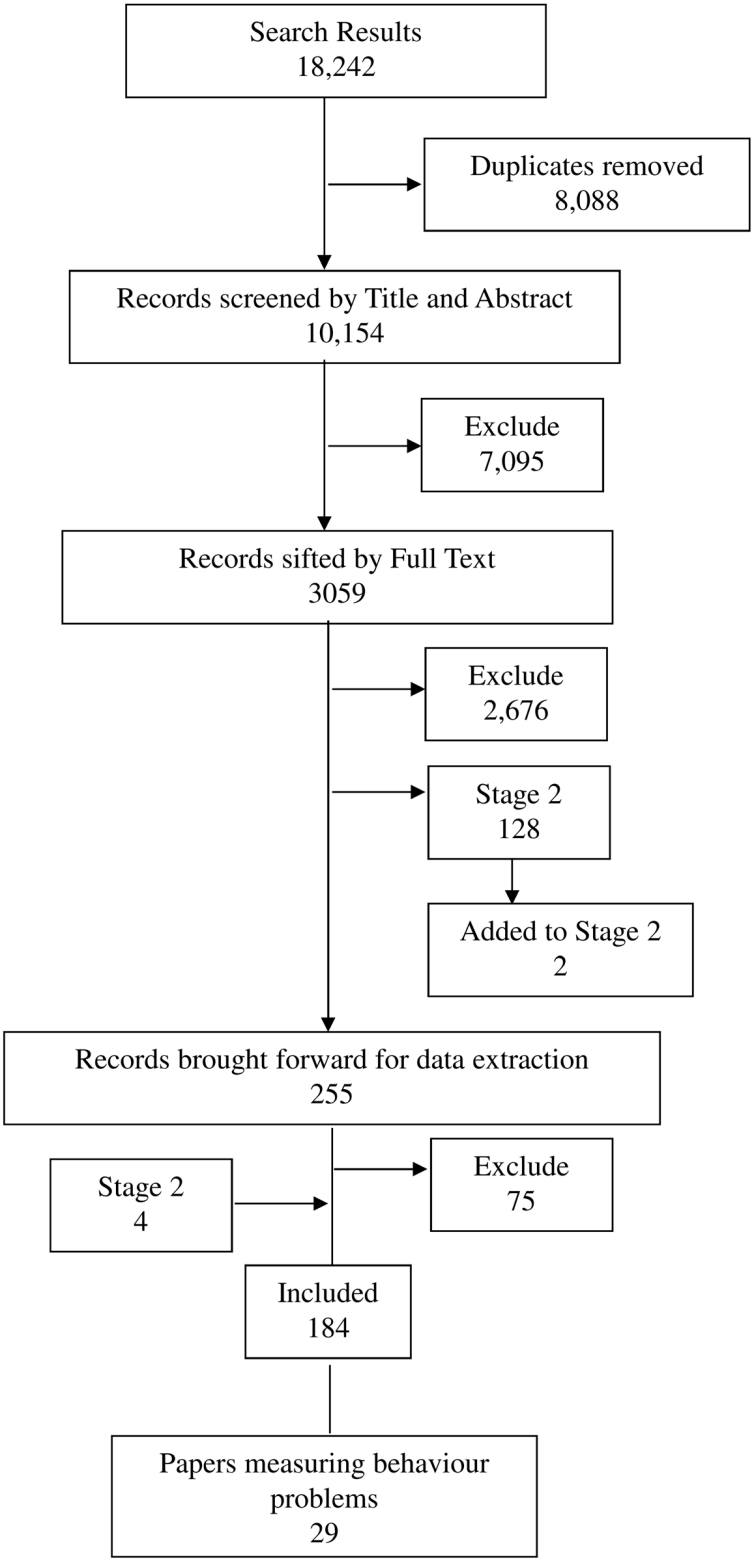
Flow Diagram of Searching and Sifting for Stage 1. Search results are up to date as of 17^th^ July 2013 (original search and update combined). Final total for data extraction = 184 (of which 29 papers included a measure of behaviour problems).

Papers were first sifted by title and abstract by one of three reviewers (see Fig 1). The decision categories were: ‘potentially include’, ‘exclude’, ‘consider for next stage’ (i.e. assesses the measurement properties of a tool only), or ‘unclear’. The three reviewers (Hanratty, Livingstone, Oono) cross-checked sets of 20 papers at a time until they reached a high level of agreement. Regular (at least weekly) discussion of decisions was held throughout the process, to maintain consistency. Then 3059 papers were examined at full text, following the same procedure above. Where decisions regarding inclusion were uncertain, a fourth reviewer (McConachie) made the final decision.

There was a further stage of sifting of records found during the search of papers about measurement properties of tools for the full review (see below Stage 2). Those searches revealed 118 records potentially relevant to Stage 1. Once duplicates were removed (86), 32 additional records were sifted by full text (completed 8^th^ December 2013); of these 28 were excluded, and four added to the final total for data extraction.

### Data extraction

Using a piloted data extraction tool the following data were extracted by one of four reviewers (Hanratty, Livingstone, Glod, Oono), with regular checks and discussion to ensure consistency; study eligibility; type of study; participant characteristics; number of outcome tools (then for each tool: name, population for which designed, specific subscales, outcomes measured according to authors). Subsequently, two reviewers with expertise in ASD (Rodgers and McConachie) reviewed each paper further and indicated which subdomains in the conceptual framework were measured by each tool including subscales. Tools were considered to measure ‘behaviour problems’ which had a primary focus on externalising behaviours which challenge others, whether or not the tool derived from a definitional framework of psychiatric disorders. (Tools with a primary focus on internalising problems such as anxiety, irritability and distress were classified under Emotional Regulation.)

### Results—Stage 1

The search identified 184 papers (see [8], of which twenty-nine measured behaviour problems [17–45] using 17 different tools (see Table 1). Twelve of these tools, reported in 24 papers, were considered further in Stage 2. Five tools (identified in five papers) were excluded because they were either developed *ad hoc* for use in particular studies [17, 21, 35], were adaptations of tools for use in another language [22], or were used only in outcome and monitoring studies published before 1995 [38] (given different diagnostic definitions for ASD before 1994).

**Table 1:** Tools used in observational and intervention studies of young children with ASD.

Tool	Paper
Aberrant Behaviour Checklist (ABC)	[18, 20, 30, 45]
Baby and Infant Screen for Children with aUtIsm Traits—Part 3 (BISCUIT-Part 3)	[40]
Behaviour Assessment System for Children, Second Edition (BASC)	[26]
Child Behaviour Checklist (CBCL)	[19, 24, 32, 41–43]
Home Situations Questionnaire (HSQ)	[20]
Nisonger Child Behaviour Rating Scales (NCBRF)	[36]
Child Behaviour Scale^x^	[27, 28]
Conners Rating Scales—Revised^x^	[23, 31, 33, 34]
Behaviour Screening Questionnaire^x^	[37]
Developmental Behaviour Checklist^x^	[25, 29, 36, 39, 44, 46]
Parent Target Problems^x^	[20]
Preschool Behaviour Checklist^x^	[37]
*Behaviour Style Questionnaire—Chinese version* *	[22]
*Coded Observation of Child Behaviour problems* **	[38]
*Functional Behaviour Assessment Interview* ***	[35]
*Parent Survey* ***	[17]
*Video coding procedures (for children and parents)* ^	[21]

^x^No evidence found at stage 2.

*non UK,

**pre-1995,

***tools developed ad hoc,

^^^observational coding.

The majority of studies were conducted in the USA (13), the UK (4), or Australia (5), with one study conducted in each of France and Holland respectively. Study designs included RCTs (4), Quasi-experimental studies (3) longitudinal (9) and cross sectional (8) observational studies. Sample sizes ranged from 16 to 762 with an age range of 18 months up to 6 years of age an overall mean age of 4 years. In 16 studies all participants had an ASD diagnosis. In the remaining 8 studies between 36% and 73% of participants had an ASD diagnosis with the other participants being either children being monitored for ASD symptoms or typically developing children.

These 12 tools, along with tools in other sub-domains, and their names and acronyms were used in Stage 2 searches, designed to identify studies of their measurement properties.

## Stage 2: Assessing the Methodological Quality of Tools for Measuring Behaviour Problems in Young Children with ASD

### Inclusion criteria—Stage 2

In stage two we looked for studies published as “full text original articles”, that evaluated tools measuring behaviour problems in samples of children that overlapped with the age range 0–6 years (e.g., a sample with age range from 6–18 was judged eligible, one that included 8–15 year olds ineligible). Studies included at least 50% children with ASD or were being monitored for ASD symptoms even if they had another primary diagnosis (e.g., a paper monitoring ASD symptoms in a Fragile X population could be eligible if exploring measurement properties of a tool used as an outcome).

We included studies of tools identified at Stage 1 (i.e. used for monitoring and/or to measure outcome in a longitudinal or intervention study with children with ASD up to 6 years old) was the focus of the study.

The aim of the study was the development of a measurement tool or the evaluation of one or more of its measurement properties. We excluded papers in which one or more of the following applied:

1. Papers in which the measurement tool was tested only for its properties in diagnostic assessment or screening and not for monitoring or measuring an outcome
2. A sample drawn only from the general population of children.
3. Sample size less than 20 (based on discussions of sample size for estimating inter-rater reliability [47] and of evidence of treatment effect [48]).
4. Studies in which the focus of the paper was not the examination of measurement properties were not eligible (for example, if the paper focused only on creating a subtype of ASD, or to group individuals by scores on the tool).
5. With regard to papers on translated tools, if the purpose was only to check the translation, then it was not eligible. If the purpose was to explore the tool’s validity in a different culture/country, and the focus was on the properties of the tool, and the findings appear relevant for use in UK, then it was included.

### Search strategy—Stage 2

Searches for Stage 2 were first conducted in March and April 2013, with iterative searches run in August, September and November 2013 and December 2014 (final search conducted on 22^nd^ December 2014). The databases searched were: ERIC; MEDLINE; EMBASE; CINAHL; PsycINFO. Again, searches were limited to English language papers only, and papers published from 1992 to present.

In order to search for papers describing studies of the measurement properties of tools, a search filter developed by the COSMIN (COnsensus-based Standards for the selection of health status Measurement INstruments) group was applied [49]. The COSMIN filter was originally designed for use in PubMed, and was translated for use in other databases by our Information Specialist (Robalino). The translation was tested in OVID, and discrepancies were discussed with Terwee (co-investigator, and part of the team who devised COSMIN). The sensitivity of the revised filters was tested continuously through the early part of data extraction, through inspection of references for ‘marker’ papers which should have been included, until the new filters were judged satisfactory. The translation can be found in S1 Text: COSMIN Translation.

Each search consisted of four components: Autism terms, age group terms, COSMIN filter, and tool name. A master search strategy was created and modified as needed for searching in various databases. Tool names required basic searches in their own right to determine variant spellings, variant names, and to include acronyms. For example, numerous tools include the word ‘scale’, but this might have been reported as ‘scales’, ‘scale’, ‘score’, or ‘scores’ by the authors. Some databases, notably PsycInfo, include a field for tests and measures, and this was utilised if available since this provides a standard way of identifying a tool regardless of how an author has reported the title.

**Fig 2:**
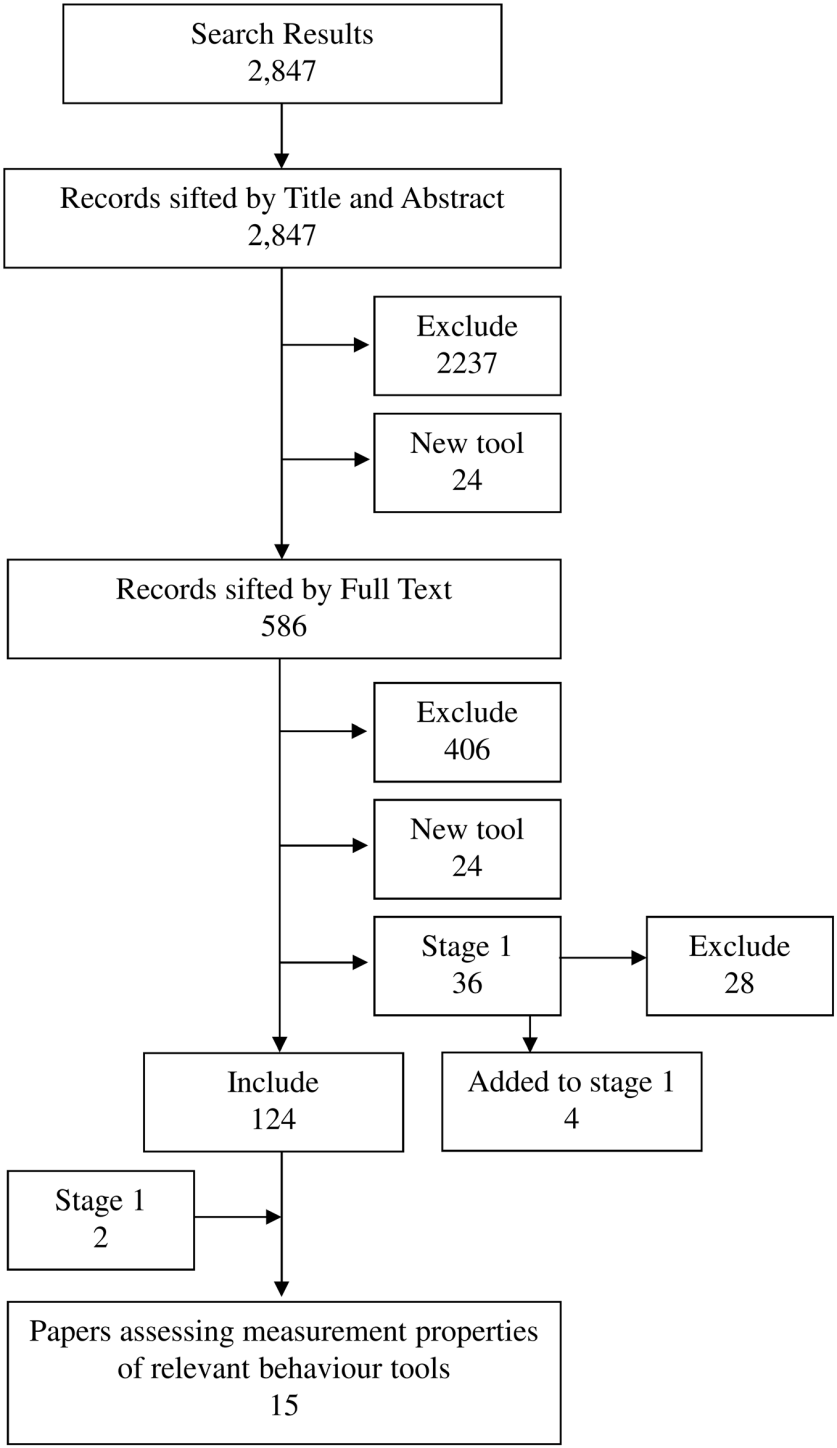
Flow Diagram of searching and sifting for Stage 2. Search Results up to date as of **22** ^**nd**^ **December 2014** (original search and update combined). Final total for data extraction = 126 of which 15 papers assessed the measurement properties of relavent behaviour tools.

Finally, the searches in Stage 1 had identified two studies [50, 51] which were about the measurement properties of identified tools for measuring behaviour problems and these were also included in Stage 2 (Fig 2).

### Selection of studies

Four reviewers (Glod, Hanratty, Livingstone, Oono) utilised the criteria to sift 10% of the articles independently and to compare results, resulting in tightening of criteria. Sifting was then conducted by a single reviewer, the team having (at random) divided up assessment of titles and abstracts, selection of full-text articles and consultation of reference lists of the studies retrieved. In case of uncertainty, the paper was discussed with McConachie before making the decision regarding inclusion. As the COSMIN rating procedure (see below) involves two stages, and the second summary stage involved a different member of the team (including McConachie) in rating the content of each article, some further exclusions were made, in a robust decision-making procedure.

### Evaluation of methodological quality

The methodological quality of Stage 2 included studies was assessed using the COSMIN checklist [52]. This checklist has 9 subscales (internal consistency, reliability, measurement error, content validity, structural validity, hypothesis testing, cross-cultural validity, criterion validity, responsiveness) with standards for how each measurement property should be assessed. Each item is scored on a four point rating scale (poor to excellent) and an overall rating for the methodological quality of each study is determined per measurement property by taking the lowest score of any item in a box (“worst score counts” method)[53].

For each study, the reviewer extracted relevant numerical and descriptive information about the properties addressed (available from the corresponding author). Terwee et al. presented criteria for judging the adequacy of each piece of information [54] (see S2 Table: Quality criteria for good measurement properties).

Ratings of study quality were then combined with the ratings of strength of the findings (see Table 2) in order to make overall judgements of each measurement tool.

**Table 2:** Levels of evidence (COSMIN).*

Level	Rating	Criteria
strong	+++ or − − −	Consistent findings in multiple studies of good methodological quality OR in one study of excellent methodological quality
moderate	++ or − −	Consistent findings in multiple studies of fair methodological quality OR in one study of good methodological quality
limited	+ or −	One study of fair methodological quality
conflicting	+/−	Conflicting findings
unknown	?	Only studies of poor methodological quality

“+” = positive rating, “?” = indeterminate rating, “−” = negative rating.

* COSMIN website: www.cosmin.nl.

### Results—Stage 2

We found no papers meeting our Stage 2 inclusion criteria for six of the 12 tools identified in Stage 1 (see Table 1). The following summarises the available evidence on the remaining six tools for which data are available on their measurement properties when used with children with ASD.

The six tools comprise four tools specifically designed for use in relation to individuals with disabilities, as well as the Behavior Assessment System for Children (BASC)—Parent Rating Scales, Second Edition [55], and two versions of the Child Behavior Checklist (CBCL)[5, 56]. The latter two tools were not designed specifically for use with disabled children and young people, but have been widely used with this population.

### Description of behaviour problems tools

**The Aberrant Behaviour Checklist** (ABC) [57] is a caregiver report checklist designed to assess maladaptive behaviours in people with developmental disabilities, from age 6 years upwards and the content derives from work with older individuals with intellectual impairments. It therefore only just overlaps with the target age group for the review. It has 58 items and is available in 40 languages. It was used in four observational studies [18, 20, 30, 45] in the review with children as young as 3 years.

**The Baby and Infant Screen for Children with aUtIsm Traits, Part 3** (BISCUIT-Part 3)[58] is a short scale of 15 items, focused on infants with Autism aged 17 to 37 months of age, to assess challenging behaviours. It presents clinical cut-off scores for no or minimal impairment, moderate impairment, and severe impairment. It was used in one observational study in the review [40].

**The Behavior Assessment System for Children Second Edition** (BASC-2)[55] is a widely used tool for assessing behaviour and emotions in children, adolescents and young adults, ranging in age from 2 to 22 years old. The BASC-2 consists of a Structured Developmental History, an Observation System, a Parent Rating Scale (134–160 items depending on age), a Self- Report of Personality Scale, and a Teacher Rating Scale (100–139 items). It was used to measure behaviour problems in one observational study in the review [26].

**The Child Behavior Checklist** (CBCL)[5, 56]. This tool is part of the Achenbach System of Empirically Based Assessment. It is a widely used tool, with two formats for children at different age bands. This is a particular strength for longitudinal studies, and both versions are included in the review. The 1.5–5 year format has 99 items, and the 6–18 years version has 118 items, with norms available for typically developing children. The items can be scored on psychiatric scales related to DSM, though this may not be relevant for children with ASD up to 6 years. It was used in three observational [19, 24, 43] and three [32, 41, 42] intervention evaluation studies in this review.

The CBCL was one of the two behaviour questionnaires presented to participants at the Discussion Day. They liked the clear instructions, with a time frame of two months, and the wide range of questions, including a qualitative section at the end enquiring about the best things about the child. However they considered that the three point scale may not provide sufficient range to capture change. The participants noted that the short questions do not establish the underlying reasons why a child might show the behaviours.

**The Home Situations Questionnaire—Pervasive Developmental Disorders version** (HSQ-PDD)[59], more recently referred to as the HSQ-ASD [60], is a 25 item caregiver questionnaire designed to assess behavioural non-compliance in everyday situations by children. It was modified from the original Home Situations Questionnaire [61] by Chowdhury et al. [59] for use in assessment of children with ASD aged 3 to 14 years, and originates from the Research Units in Pediatric Psychopharmacology Autism Network. The HSQ-PDD was used in one intervention evaluation study [20] in the review.

**The Nisonger Child Behavior Rating Form** (NCBRF)[62] is a rating scale designed to assess social competence and problem behaviour in children with developmental disabilities. There are parent and teacher versions of the scale, which has 76 items altogether, with 10 positive social items before the 66 problem items. Parents are also invited to mention special circumstances which may have affected the child’s behaviour in the last month. The age range for the NCBRF is 3 to 16 years.

This tool was also examined by participants at the Discussion Day, who particularly appreciated that the items included some which were relevant to ASD. However, participants thought some items were poorly worded (e.g. “resisted provocation”), several were not relevant to children in the age range up to 6 years (including items such as “feels worthless or inferior”) and some items would be typical for a 3 year old (e.g. “runs away from adults”). This tool was used in one intervention evaluation study [36] in the review.

### Measurement properties

The searches identified 15 papers that assessed one or more of the measurement properties of these six tools [50, 51, 58, 59, 63–73]. Table 3 details the evidence found for each tool and Table 4 summarises the overall strength and quality of evidence relating to each of the tools. (See section on ‘Evaluation of methodological quality’ above.)

**Table 3:** Quality and direction of evidence for measurement properties of included tools measuring behaviour problems.

			Reliability				Hypothesis testing			
Measure	Article	Internal Consistency	Test-retest	Inter-rater	Content Validity	Structural Validity	Convergent /divergent	Known groups	Criterion Validity	Responsiveness
ABC	Brinkley 2007[64]					excellent +				
	Karabekiroglu 2009[50]	fair +						fair +	fair +	
	Sigafoos 1997[73]					poor +				
	Kuhlthau 2013[67]						fair +			
	Kaat 2014[66]	good +				good -	good +		good +	
BISCUIT-Part 3	Matson 2009a [58]	good +								
	Matson 2009b [71]	excellent +				excellent -				
BASC-2	Hass 2010[65]	fair +						fair +		
	Mahan 2011[70]							good -		
CBCL 1.5–5	Pandolfi 2009[72]	good +				good +				
CBCL 6–18	Pandolfi 2012[51]	good +				good +			good +	
	Kuhlthau 2013[67]						fair -			
HSQ- PDD version	Chowdhury 2010[59]	excellent +				excellent +		excellent +		excellent +
	Arnold 2012[63]									good +
NCBRF	Lecavalier 2004[68]	good +				good -				
	Lecavalier 2006[69]		fair +	fair -				fair +		

**“+”** = positive rating; “–” = negative rating.

**Table 4:** Summary of quality of tools for measuring behaviour problems in children with ASD.

		Reliability				Hypothesis testing			
Tool	Internal Consistency	Test-retest	Inter rater	Content Validity	Structural Validity	Convergent/ divergent validity	Known groups	Criterion Validity	Responsiveness
Aberrant Behaviour Checklist	++				+++	++	+	++	
Baby and Infant Screen for Children with aUtIsm Traits—Part 3	+++				− − −				
Behavior Assessment System for Children—Second Edition	+						+/−		
Child Behavior Checklist 1.5–5	++				++				
Child Behavior Checklist 6–18	++				++	−		++	
Home Situations Questionnaire-Pervasive Developmental Disorders version	+++				+++	+++			+++
Nisonger Child Behavior Rating Form	++	+	−		− −	+			

“+++” or “− − −” = strong evidence, “++” or “− −” = moderate evidence, “+” or “−” = limited evidence, “?” = unknown, due to poor methodological quality, blank cell = no evidence available.

#### Aberrant Behaviour Checklist (ABC)

The ABC [57] items are scored in five subscales: Irritability, Lethargy/Social Withdrawal, Stereotypic Behavior, Hyperactivity/Non-compliance, and Inappropriate speech. Internal consistency was reported as good by Karabekiroglu and Aman [50] (Cronbach’s alphas from 0.68 to 0.90) and by Kaat, Lecavalier and Aman [66] (alphas from .77 to .94). Inter-rater reliability (between similar raters) and test-retest reliability were not assessed. Brinkley et al. [64] and Kaat, Lecavalier and Aman [66] demonstrated that the ABC had good structural validity; the latter very large study (n = 1893) found that 90% of items matched the standard ABC factor structure, though model fit was ‘marginal’ (Root Mean Square Error of Approximation (RMSEA) was .086). Sigafoos et al. [73] also showed that the ABC had good structural validity with five factors, though due to the small sample size (n = 32), the Sigafoos paper was judged to be of poor methodological quality. Karabekiroglu and Aman [50] showed that the ABC distinguished between clinical subgroups. Kaat, Lecavalier and Aman [66] found as expected that irritability and hyperactivity decreased with age. Kaat, Lecavalier and Aman [66] and Karabekiroglu and Aman [50] found predicted significant correlations with related constructs measured by the Child Behaviour Checklist and the Autism Behaviour Checklist, and Kuhlthau et al [67] with a measure of child quality of life, the Child Health and Illness Profile—Child Edition.

#### Baby and Infant Screen for Children with aUtIsm Traits—Part 3 (BISCUIT-Part 3)

The BISCUIT-Part 3 [58] items are organised into three subscales: Aggressive/Disruptive behaviours, Stereotypic behaviours, and Self-Injurious behaviour. Internal consistency of the BISCUIT-Part 3 was reported as good with Cronbach’s alpha >0.70 in two papers [58, 71] but reliability was not assessed. Structural validity, assessed in Matson, Boisjoli et al [71] was not acceptable, with the exploratory factor analysis resulting in a three factor solution explaining just 38.32% of the variance.

#### Behavior Assessment System for Children Second Edition (BASC-2)

The BASC-2 Parent and Teacher Rating Scale [55] items are organised into 9 clinical subscales: Aggression, Anxiety, Attention problems, Atypicality, Conduct problems, Depression, Hyperactivity, Somatization, Withdrawal (as well as five adaptive scales). Hass et al. [65] showed that the BASC-2 had acceptable internal consistency for the 10 item aggression scale and the 9 item conduct problem scale with teachers as informants. There were also significant large differences between children with ASD and matched controls on the aggression scale (Cohen’s d = 0.58) and the externalising problems composite scale (Cohen’s d = 0.75). Mahan and Matson [70] also assessed known groups validity of the BASC-2, with parents as informants. ASD children scored significantly greater than typically developing children on the conduct problems and externalising composite scales, but did not differ as expected on the aggression subscale. No evidence was found on reliability, structural validity or criterion validity for the clinical scales.

#### Child Behavior Checklist (CBCL) 1.5–5

CBCL 1.5–5 year [5] subscale scores are derived for the following syndromes: Emotionally Reactive, Anxious/Depressed, Somatic Complaints, Withdrawn, Sleep Problems, Attention problems, and Aggressive Behaviour, and these are further summed to provide scores for Internalizing and Externalizing problems.

The CBCL 1.5–5 was assessed by one paper of good methodological quality [72] with a sample of children with ASD. This paper provided evidence of good internal consistency for total problems (Cronbach’s alpha = 0.93) and both the externalizing behaviour domain (Cronbach’s alpha = 0.90) and aggressive behaviour sub-scale (Cronbach’s alpha = 0.80). No evidence was found concerning reliability. Structural validity was also good with acceptable model fit for a one factor model for aggressive behaviour (RMSEA<0.06, Comparative Fit Index (CFI)>0.95) indicating that there was a single latent factor underlying this sub-scale.

#### Child Behavior Checklist 6–18

The CBCL 6–18 [56] was assessed with a sample of ASD youth in two papers [51, 67]. Pandolfi, Magyar and Dill [51] found internal consistency was good with r = 0.92 for the aggressive behaviour scale, but no evidence was found concerning reliability. Structural validity for the complete measure was good and analysis supported the original two factor structure of the CBCL 6–18 (internalizing and externalising factors). Tests of unidimensionality of scales did not reach the cut off for acceptable fit for aggressive behaviour (RMSEA = 0.10, CFI = 0.95); however, convincing arguments were provided to allow for correlated disturbances in the model for two item pairs (destroys own things/destroys others things and disobedient at home/disobedient at school). This adjusted model demonstrated acceptable fit (RMSEA<0.06, CFI>0.95). Criterion validity was assessed by Pandolfi, Magyar and Dill [51] by comparing ASD children with and without a co-occurring emotional/behavioural difficulty. Children with a co-occurring EBD scored significantly higher than those without EBDs on total problems, though there were no significant differences between the two groups for aggressive behaviour or externalising behaviour as the most common co-occurring EBDs in the sample were anxiety disorders. Kuhlthau et al [67] hypothesised that externalising behaviours would be more strongly associated with quality of life than internalising behaviours in children with ASD, but this was not supported.

#### Home Situations Questionnaire-Pervasive Developmental Disorders version (HSQ-PDD)

The HSQ-PDD [59] items are scored in two subscales: Socially Inflexible, and Demand-Specific. The properties of the HSQ-PDD were assessed in a sample of 124 children aged 4 to 13 years. Structural validity for a two factor solution was a reasonable fit (RMSEA 0.06) and internal consistency good (alpha 0.90 for the ‘socially inflexible’ subscale and 0.80 for ‘demand-specific’). Known groups validity and responsiveness (change over time) were also shown as good for the HSQ-PDD by Chowdhury et al [59]. In a further paper, responsiveness was shown related as hypothesised to change in the Vineland Daily Living Skills scale [63].

#### Nisonger Child Behavior Rating Form (NCBRF)

The NCBRF [62] has six problem behaviour subscales: Conduct Problem, Insecure/Anxious, Hyperactive, Self-Injury/Stereotypic, Self-isolated/Ritualistic, and Overly Sensitive. Internal consistency of the problem behaviour scales was reported as good with Cronbach’s alpha >0.70 for all subscales in both parent and teacher versions [68]. Test-retest reliability for the parent version was reported to be strong (ICC for total problem behaviour >0.80) but the teacher version fell short of the COSMIN criterion (ICC for total problem behaviour = 0.68); however, over a one year time interval some change might well be expected. Agreement was low between parents and teachers on common items from the parent and teacher versions of the scale, indicating that inter-rater reliability was poor [69]. Structural validity was also shown to be poor for problem behaviour with a 5 factor solution accounting for 47.5% of the variance [68]. Finally, Lecavalier, Leone & Wiltz [69] provided fair evidence for divergent and convergent validity of the NCBRF though criterion validity was not assessed.

## Discussion

The evidence presented here on the measurement properties of six tools used to measure behaviour problems in young children with ASD is good in parts, and strikingly thin in others.

Firstly, for these six tools there is little evidence on reliability, and where present the quality of the evidence is ‘fair’ at best. There is a need to know how much variability there may be in adult reports of children’s behaviour when making judgements about the significance of change over time.

Secondly, the difficulties for researchers in designing studies to establish whether tools are sensitive to change are crucially dependent on both intervention and measurement. That is, it is necessary to have clear evidence of treatment effect on one responsive tool in order to test whether a new tool is also responsive. Only the HSQ-PDD team have considered this question explicitly within a treatment study. Such attention to responsiveness in the development of a tool is important in strengthening the evidence-base for particular treatment approaches (e.g. parent training for disruptive behaviours [74]).

Thirdly, the evidence for the structural validity of tools was somewhat mixed, with factor analyses presented for the BISCUIT-Part 3 and the NCBRF accounting for rather low proportions of the variance in the data.

A fourth weakness is the lack of reported ASD-specific work on content validity, particularly in the case of the CBCL and BASC-2 designed originally for typical populations. A source of difficulty in evaluation of measurement tools is the challenge in separating out the measurement of autism characteristics from other problems measured, such as behaviour problems or anxiety [75]. In the case of the CBCL, Hus and colleagues [76] have shown that higher Social Responsiveness Scale scores (measuring ASD characteristics) were associated with greater behaviour problems in 2368 children with ASD, mean age 8 years, and in their non-affected siblings. The authors concluded that report of the severity of ASD characteristics may be influenced by factors that are not specific to ASD. However, equally it seems that behaviours such as ‘doesn’t answer when people talk to him/her’ or ‘strange behavior’ are labeled as ‘behaviour problems’ in the questionnaires when their primary characteristic appears to relate to ASD.

Overall, in terms of strength of measurement properties the choice appears to fall between the CBCL and the HSQ-PDD. The CBCL was not designed for children with disability and lack of evidence on content validity in use with children with ASD is a weakness; however, the existence of compatible forms across a wide age range is a strength. The availability of norms may also be of use (however, see comments above concerning content validity). Further evidence on test-retest and inter-rater reliability, and sensitivity to change, when used with young children with ASD would be valuable. The HSQ-PDD is a relatively new tool, and the team are continuing to explore the strongest grouping of items [77]. Further evidence of its measurement properties, by teams other than the original developers, will strengthen conclusions about its usefulness in future.

### Strengths and limitations of the evidence

This systematic review had some limitations of method, notably the restriction of studies to those reported in English. The restricted age range for the review—the measurement properties of tools used with children with ASD up to the age of six years—can be considered a limitation where researchers wish for evidence on the robustness of tools used to measure outcomes in older individuals on the autism spectrum (e.g. drug trials, interventions in education settings). Nevertheless, the evidence presented is intended to offer guidance to those conducting psycho-social interventions with preschool children. A further limitation is that the two-stage search process will have missed examination of tools used in more recent early intervention evaluation and observational studies. However, the strengths of the review lie in the wide search strategies utilised, and a team of reviewers working together consistently.

The findings of the review have been hampered by a lack of articles identified which specifically consider measurement properties of tools in use with children with ASD. We had intended to extract information about the reliability, validity and responsiveness to change of tools as described in the intervention evaluation and observational studies (Stage 1), but most studies simply cited the reliability and validity of tools from their source references, irrespective of whether this had been tested with samples of children with ASD. Furthermore, it was not possible to interpret the evidence on responsiveness to change without considering whether the study was adequately powered to detect change, and whether the choice of outcome tool was appropriate to the nature of the intervention. If a significant intervention effect was not shown, there were a number of possible reasons, and the properties of the tool constituted only one of those reasons. Therefore, the decision was taken to rely only on the systematic assessment of measurement properties of tools described in Stage 2 for the evaluation.

### Other approaches to measurement of behaviour problems

Six tools were found at Stage 1 for which no articles appeared to have considered their measurement properties in use with children with ASD. One of these was an approach which individualises assessment for children, ‘Target Behaviours’. With the individuality of needs of young children with ASD, it may be particularly appropriate to adopt an idiographic approach to outcome measurement such as this, or Goal Attainment Scaling (GAS). Although the focus is individual, the scoring systems enable comparison across individuals. The Target Behaviours (or target symptoms) methodology was included in the battery of tools recommended by the Research Units on Pediatric Psychopharmacology [78] and used by one study in this review [20]. Where a specific behaviour is the target of intervention, the parent is interviewed about its nature, frequency and intensity, and a vignette description is prepared. At follow-up the same questions are asked about the behaviour; the two vignettes are then compared and rated for degree of change on a 9 point scale by an expert panel. Thus this idiographic measure allows for ‘blind’ rating, and provides an opportunity to capture change. Inter-rater reliability across the expert panel can be assessed. GAS requires greater professional input (than Target Behaviours), including training and practice, to enable a suitable behavioural goal to be defined and scaled (with description of outcomes on a 5 point scale between ‘worst expected outcome’ to ‘best expected outcome’). There are continuing debates about appropriate statistical analyses of GAS scores, such as whether accomplishment of different individual goals can be summed into a group score. Nevertheless if the GAS scores are done by observation, the assessor can be ‘blind’ [79]. These approaches to responsive measurement of relevant and individualised outcomes merit further exploration for young children with ASD.

In the process of the final sifting for Stage 2, four other ‘new’ tools were identified that measured behaviour problems and had been used to describe young children with ASD, but not in the intervention and observation studies searched for in Stage 1. These are the Behaviour Function Inventory [80], the Behavior Problems Inventory [81] (and its Short form [46]), the Children’s Scale of Hostility and Aggression: Reactive/Proactive [82], and the Child’s Challenging Behaviour Scale [83]. Future updates of the review will, hopefully, include evidence about these tools and their use in outcome measurement in evaluation and observation studies with young children with ASD. As the requirements for establishing the measurement properties of a tool become more standardised [84] and better understood, it is anticipated that the quality of the available evidence will be higher.

In summary, despite the strong likelihood of problem behaviours in young children with ASD, and consequently the need for effective intervention approaches, there are significant limitations in the measurement tools currently in use in intervention evaluation and observational studies. The paper identifies the strongest candidate measurement tools to be used in future studies, and suggests the gaps in knowledge which require to be filled.

## Supporting Information

S1 PRISMA Checklist PRISMA checklist. (DOC)

S1 Table Search strategy. (DOCX)

S2 Table Quality criteria for good measurement properties. (DOCX)

S1 Text COSMIN translation. (DOCX)
