# Supplementary material for: Systematic Review of the Measurement Properties of Tools Used to Measure Behaviour Problems in Young Children with Autism
Source: PLoS One. 2015 Dec 14;10(12):e0144649. doi: 10.1371/journal.pone.0144649 (PMC4689504; doi:10.1371/journal.pone.0144649)
Supplement: S1 Table — (DOCX) [file pone.0144649.s002.docx]

**S1 Table: Search Strategy**

Master Search Strategy stage 1 and sample from MEDLINE

**Search terms**

| **AUTISM TERMS** | **AGE GROUP TERMS** |
| --- | --- |
| ASC | Child* |
| ASD (NOT atrial septal defect) | elementary (school) |
| Asperger* | infan* |
| Autis* | kindergarten* |
| childhood schizophrenia | nursery |
| communicat* | p?ediatric* |
| Kanner* | pre-school* |
| language delay* | preschool* |
| PDD | primary (school) |
| pervasive developmental disorder | toddler* |
| speech disorder* | special needs |
| semantic-pragmatic disorder | grammar (school) |
| PDD-NOS | exp child/ [MeSH] |
| exp Child Development Disorders, Pervasive/ [MeSH] |  |
| **ASSESSMENT-RELATED TERMS** | **BEHAVIOUR-RELATED TERMS** |
| Assess* | Behavio?r |
| exam* | intervention* |
| feasib* | non-verbal |
| measur* | program* |
| method* | rehabilitat* |
| questionnaire* | social interaction |
| reliab* | therap* |
| repeat* | train OR training OR trained |
| report* | treatment* |
| reproducib* | verbal |
| self-report* |  |
| survey* |  |
| test* |  |
| valid* |  |
| score* |  |
| diagnostic* |  |
| observ* |  |
| track* |  |
| monitor* |  |
| follow-up |  |
| scale |  |
| outcome* |  |
| audit* |  |
| record* |  |

**Example search strategy**

**MEDLINE (OVID)**

1. (asd not atrial septal defect).ab,ti.

2. "Asperger*".ab,ti.

3. "Autis*".ab,ti.

4. childhood schizophrenia.ab,ti.

5. "Kanner*".ab,ti.

6. (PDD or PDD-NOS).ab,ti.

7. semantic-pragmatic disorder.ab,ti.

8. "language delay*".ab,ti.

9. "speech disorder*".ab,ti.

10. pervasive developmental disorder.ab,ti.

11. exp Child Development Disorders, Pervasive/

12. or/1-11

13. exp Child/

14. "infan*".ab,ti.

15. "child*".ab,ti.

16. "kindergarten*".ab,ti.

17. nursery.ab,ti.

18. "p?ediatric*".ab,ti.

19. (pre-school* or preschool*).ab,ti.

20. "toddler*".ab,ti.

21. special needs.ab,ti.

22. ((primary or elementary or grammar) and school).ab,ti.

23. or/13-22

24. 12 and 23

25. Behavio?r.tw.

26. "intervention*".tw.

27. non-verbal.tw.

28. program$4.tw.

29. "rehabilitat*".tw.

30. social interaction.tw.

31. "therap*".tw.

32. "treatment*".tw.

33. verbal.tw.

34. (train or training or trained).tw.

35. or/25-34

36. 24 and 35

37. limit 36 to (english language and humans and yr="1992 -Current")

38. "assess*".tw.

39. "feasib*".tw.

40. "measur*".tw.

41. "method*".tw.

42. "questionnaire*".tw.

43. "reliab*".tw.

44. "repeat*".tw.

45. "report*".tw.

46. "reproducib*".tw.

47. "self-report*".tw.

48. "survey*".tw.

49. "valid*".tw.

50. "score*".tw.

51. "diagnostic*".tw.

52. "observ*".tw.

53. "track*".tw.

54. "monitor*".tw.

55. follow-up.tw.

56. scale.tw.

57. "outcome*".tw.

58. "audit*".tw.

59. "record*".tw.

60. or/38-59

61. 37 and 60

62. limit 61 to (english language and humans and yr="1992 -Current")

63. Epidemiologic Studies/

64. 62 and 63

65. cohort.ti,ab. or exp Cohort Studies/ or longitudinal.ti,ab. or prospective.ti,ab. or retrospective.ti,ab.

66. 62 and 65

67. exp Clinical Trial/ or double-blind method/ or (clinical trial* or randomized controlled trial or multicenter study).pt. or exp Clinical Trials as Topic/ or ((randomi?ed adj7 trial*) or (controlled adj3 trial*) or (clinical adj2 trial*) or ((single or doubl* or tripl* or treb*) and (blind* or mask*))).ti,ab.

68. limit 67 to yr="1992-2012"

69. 62 and 68

70. ((("semi-structured" or semistructured or unstructured or informal or "in-depth" or indepth or "face-to-face" or structured or guide) adj3 (interview* or discussion* or questionnaire*)) or (focus group* or qualitative or ethnograph* or fieldwork or "field work" or "key informant")).ti,ab. or interviews as topic/ or focus groups/ or narration/ or qualitative research/

71. 62 and 70

72. ((systematic adj3 literature) or systematic review* or meta-analy* or metaanaly* or "research synthesis" or ((information or data) adj3 synthesis) or (data adj2 extract*)).ti,ab. or (cinahl or (cochrane adj3 trial*) or embase or medline or psyclit or (psycinfo not "psycinfo database") or pubmed or scopus or "sociological abstracts" or "web of science").ab. or "cochrane database of systematic reviews".jn. or ((review adj5 (rationale or evidence)).ti,ab. and review.pt.) or meta-analysis as topic/ or Meta-Analysis.pt.

73. 62 and 72

74. limit 73 to yr="1992 -Current"

75. exp Case-Control Studies/ or Control Groups/ or Matched-Pair Analysis/ or ((case* adj5 control*) or (case adj3 comparison*) or control group*).ti,ab.

76. 62 and 75

77. 64 or 66 or 69 or 71 or 74 or 76
