# Supplementary material for: Systematic Review of the Measurement Properties of Tools Used to Measure Behaviour Problems in Young Children with Autism
Source: PLoS One. 2015 Dec 14;10(12):e0144649. doi: 10.1371/journal.pone.0144649 (PMC4689504; doi:10.1371/journal.pone.0144649)
Supplement: S2 Table — (DOCX) [file pone.0144649.s003.docx]

**S2 Table:** Quality criteria for good measurement properties*

| **Property** | **Rating** | **Quality Criteria** |
| --- | --- | --- |
| **Reliability** |  |  |
| Internal consistency | **+** | Cronbach's alpha(s) ≥ 0.70 |
|  | **?** | Cronbach's alpha not determined or dimensionality unknown |
|  | **-** | Cronbach's alpha(s) < 0.70 |
| Reliability | **+** | ICC / weighted Kappa ≥ 0.70 OR Pearson’s r ≥ 0.80 |
|  | **?** | Neither ICC / weighted Kappa, nor Pearson’s r determined |
|  | **-** | ICC / weighted Kappa < 0.70 OR Pearson’s r < 0.80 |
| Measurement error | **+** | MIC > SDC OR MIC outside the LOA |
|  | **?** | MIC not defined |
|  | **-** | MIC ≤ SDC OR MIC equals or inside LOA |
| **Validity** |  |  |
| Content validity | **+** | All items are considered to be relevant for the construct to be measured, for the target population, and for the purpose of the measurement AND the questionnaire is considered to be comprehensive |
|  | **?** | Not enough information available |
|  | **-** | Not all items are considered to be relevant for the construct to be measured, for the target population, and for the purpose of the measurement OR the questionnaire is considered not to be comprehensive |
| Construct validity or Structural validity | **+** | EFA: Factors should explain at least 50% of the variance; CFA: RMSEA ≤0.06, CFI or TLI ≥ 0.95. |
|  | **?** | Explained variance not mentioned |
|  | **-** | EFA: Factors explain < 50% of the variance; CFA: RMSEA >0.06, CFI or TLI < 0.95 |
| Hypothesis testing | **+** | Correlations with instruments measuring the same construct ≥ 0.50 OR at least 75% of the results are in accordance with the hypotheses AND correlations with related constructs are higher than with unrelated constructs |
|  | **?** | Solely correlations determined with unrelated constructs |
|  | **-** | Correlations with instruments measuring the same construct < 0.50 OR  < 75% of the results are in accordance with the hypotheses OR correlations with related constructs are lower than with unrelated constructs |
| Criterion validity | **+** | Convincing arguments that gold standard is “gold” AND correlation with gold standard ≥ 0.70 |
|  | **?** | No convincing arguments that gold standard is “gold” OR doubtful design or method |
|  | **-** | Correlation with gold standard < 0.70, despite adequate design and method |
| **Responsiveness** |  |  |
| Responsiveness | **+** | Correlation with changes on instruments measuring the same construct ≥ 0.50 OR at least 75% of the results are in accordance with the hypotheses OR AUC ≥ 0.70 AND correlations with changes in related constructs are higher than with unrelated constructs |
|  | **?** | Solely correlations determined with unrelated constructs |
|  | **-** | Correlations with changes on instruments measuring the same construct < 0.50 OR < 75% of the results are in accordance with the hypotheses OR AUC < 0.70 OR correlations with changes in related constructs are lower than with unrelated constructs |

MIC = minimal important change, SDC = smallest detectable change, LoA = limits of agreement, ICC = intraclass correlation coefficient, AUC = area under the curve. + = positive rating; ? = indeterminate rating; - = negative rating *COSMIN website: [www.cosmin.nl](http://www.cosmin.nl)
