# Supplementary material for: Systematic Review of the Measurement Properties of Tools Used to Measure Behaviour Problems in Young Children with Autism
Source: PLoS One. 2015 Dec 14;10(12):e0144649. doi: 10.1371/journal.pone.0144649 (PMC4689504; doi:10.1371/journal.pone.0144649)
Supplement: S1 Text — (DOCX) [file pone.0144649.s004.docx]

**S1 Text: COSMIN translation (for OVID) and measurement tool search strategy**

instrumentation.sh. OR methods.sh. OR Validation Studies.pt. OR Comparative Study.pt. OR psychometrics/ OR psychometr*.ab,ti. OR clinimetr*.tw. OR clinometr*.tw. OR "Outcome Assessment (Health Care)"/ OR outcome assessment.ab,ti. OR outcome measure*.tw. OR observer variation/ OR observer variation.ab,ti. OR Health Status Indicators/ OR reproducibility of results/ OR reproducib*.ab,ti. OR discriminant analysis/ OR reliab*.ab,ti. OR unreliab*.ab,ti. OR valid*.ab,ti. OR coefficient.ab,ti. OR homogeneity.ab,ti. OR homogeneous.ab,ti. OR internal consistency.ab,ti. OR (cronbach*.ab,ti. AND (alpha.ab,ti. OR alphas.ab,ti.)) OR (item.ab,ti. AND (correlation*.ab,ti. OR selection*.ab,ti. OR reduction*.ab,ti.)) OR agreement.ab,ti. OR precision.ab,ti. OR imprecision.ab,ti. OR precise values.ab,ti. OR test-retest.ab,ti. OR (test.ab,ti. AND retest.ab,ti.) OR (reliab*.ab,ti. AND (test.ab,ti. OR retest.ab,ti.)) OR stability.ab,ti. OR interrater.ab,ti. OR inter-rater.ab,ti. OR intrarater.ab,ti. OR intra-rater.ab,ti. OR intertester.ab,ti. OR inter-tester.ab,ti. OR intratester.ab,ti. OR intra-tester.ab,ti. OR interobserver.ab,ti. OR inter-observer.ab,ti. OR intraobserver.ab,ti. OR intraobserver.ab,ti. OR intertechnician.ab,ti. OR inter-technician.ab,ti. OR intratechnician.ab,ti. OR intra-technician.ab,ti. OR interexaminer.ab,ti. OR inter-examiner.ab,ti. OR intraexaminer.ab,ti. OR intra-examiner.ab,ti. OR interassay.ab,ti. OR inter-assay.ab,ti. OR intraassay.ab,ti. OR intra-assay.ab,ti. OR interindividual.ab,ti. OR inter-individual.ab,ti. OR intraindividual.ab,ti. OR intra-individual.ab,ti. OR interparticipant.ab,ti. OR inter-articipant.ab,ti. OR intraparticipant.ab,ti. OR intra-participant.ab,ti. OR kappa.ab,ti. OR kappa’s.ab,ti. OR kappas.ab,ti. OR repeatab*.ab,ti. OR ((replicab*.ab,ti. OR repeated.ab,ti.) AND (measure.ab,ti. OR measures.ab,ti. OR findings.ab,ti. OR result.ab,ti. OR results.ab,ti. OR test.ab,ti. OR tests.ab,ti.)) OR generaliza*.ab,ti. OR generalisa*.ab,ti. OR concordance.ab,ti. OR (intraclass.ab,ti. AND correlation*.ab,ti.) OR discriminative.ab,ti. OR known group.ab,ti. OR factor analysis.ab,ti. OR factor analyses.ab,ti. OR dimension*.ab,ti. OR subscale*.ab,ti. OR (multitrait.ab,ti. AND scaling.ab,ti. AND (analysis.ab,ti. OR analyses.ab,ti.)) OR item discriminant.ab,ti. OR interscale correlation*.ab,ti. OR error.ab,ti. OR errors.ab,ti. OR individual variability.ab,ti. OR (variability.ab,ti. AND (analysis.ab,ti. OR values.ab,ti.)) OR (uncertainty.ab,ti. AND (measurement.ab,ti. OR measuring.ab,ti.)) OR standard error of measurement.ab,ti. OR sensitiv*.ab,ti. OR responsive*.ab,ti. OR ((minimal.ab,ti. OR minimally.ab,ti. OR clinical.ab,ti. OR clinically.ab,ti.) AND (important.ab,ti. OR significant.ab,ti. OR detectable.ab,ti.) AND (change.ab,ti. OR difference.ab,ti.)) OR (small*.ab,ti. AND (real.ab,ti. OR detectable.ab,ti.) AND (change.ab,ti. OR difference.ab,ti.)) OR meaningful change.ab,ti. OR ceiling effect.ab,ti. OR floor effect.ab,ti. OR Item response model.ab,ti. OR IRT.ab,ti. OR Rasch.ab,ti. OR Differential item functioning.ab,ti. OR DIF.ab,ti. OR computer adaptive testing.ab,ti. OR item bank.ab,ti. OR cross-cultural equivalence.ab,ti.
